# Carbon Dioxide is a Powerful Inducer of Monokaryotic Hyphae and Spore Development in *Cryptococcus gattii* and Carbonic Anhydrase Activity is Dispensable in This Dimorphic Transition

**DOI:** 10.1371/journal.pone.0113147

**Published:** 2014-12-05

**Authors:** Ping Ren, Vishnu Chaturvedi, Sudha Chaturvedi

**Affiliations:** 1 Mycology Laboratory, Wadsworth Center, New York State Department of Health, 120 New Scotland Avenue, Albany, New York, United States of America; 2 Department of Biomedical Sciences, School of Public Health, State University at New York, Albany, New York, United States of America; Yonsei University, Republic Of Korea

## Abstract

*Cryptococcus gattii* is unique among human pathogenic fungi with specialized ecological niche on trees. Since leaves concentrate CO_2_, we investigated the role of this gaseous molecule in *C. gattii* biology and virulence. We focused on the genetic analyses of β-carbonic anhydrase (β-CA) encoded by *C. gattii CAN1* and *CAN2* as later is critical for CO_2_ sensing in a closely related pathogen *C. neoformans*. High CO_2_ conditions induced robust development of monokaryotic hyphae and spores in *C. gattii*. Conversely, high CO_2_ completely repressed hyphae development in sexual mating. Both *CAN1* and *CAN2* were dispensable for CO_2_ induced morphogenetic transitions. However, *C. gattii CAN2* was essential for growth in ambient air similar to its reported role in *C. neoformans*. Both *can1* and *can2* mutants retained full pathogenic potential *in vitro* and *in vivo*. These results provide insight into *C. gattii* adaptation for arboreal growth and production of infectious propagules by β-CA independent mechanism(s).

## Introduction

*Cryptococcus gattii*, a basidiomycetous yeast, is an emerging pathogen in North America causing fatal disease in both healthy and immunocompromised humans as well in a wide range of animals including birds, domestic and wild mammals [1], [2]. A large outbreak of *C. gattii* infection among humans and animals in Vancouver Island, British Columbia, Canada, and the isolation of *C. gattii* from several genera of trees other than *Eucalyptus*, have indicated that this fungus must have broader geographic distribution including Pacific Northwest in the United States, and around the world [1], [3]–[8]. In extensive ecological investigations, *C. gattii* was isolated readily from soil, air, and water surrounding trees, in regions in the vicinity of Vancouver Island; evidently, *C. gattii* dispersal in the environment has been occurring through distribution of tree byproducts, aerosolization, water flow, and arthropogenic factors [9], [10].

Given the numerous possibilities for *C. gattii* dispersal, the organism’s *de novo* colonization mechanisms on trees and regions surrounding these trees are far from clear. Xue *et al* [11] have demonstrated that the young *Arabidopsis thaliana* plant surfaces represent a permissible environment, in which *C. gattii* and its closely related species *C. neoformans* can complete their sexual cycle (**α-a** mating). This intriguing finding raised the possibility that plants might serve as a critical host in the production of infectious propagules in the form of sexual spores (basidiospores). However, the predominance of α mating type both clinically and environmentally indicated that sexual mating in nature might be a limited and rare event. A number of studies raised the possibility that monokaryotic fruiting (**α-α** mating or same sex mating) might be a widespread phenomenon in *C. neoformans* var. *neoformans*, *C. neoformans* var. *grubii* and *C. gattii* [12]–[14]. Studies examining strains from outbreak investigations on Vancouver Island have found diploid isolates of α mating type with heterozygous alleles at their **α-** mating locus suggesting that the hypervirulent *C. gattii* VGII outbreak strains arose as a result of **α**-**α** mating [15]. Interestingly, the fruiting body (basidium) containing basidiospores as a result of **α**-**α** mating were not observed in *C. gattii* in the laboratory setting [16]. Therefore, it is possible that monokaryotic fruiting results from mating-dependent and mating-independent developmental pathways. A recent study from *C. neoformans* var. *neoformans* found cell cycle arrest induced mating-independent monokaryotic fruiting[17].

*C. gattii* is unique among human pathogenic fungi in its ecological niche; it predominantly inhabits trees by mechanisms not yet clearly understood. Since plants concentrate CO_2_ through the action of Ribulose-1,5-bisphosphate carboxylase/oxygenase (RubisCo), it is conceivable that *C. gattii* is sensing CO_2_ for its survival and propagation in the environment [18]. A number of reports provide insight into how pathogenic fungi sense environmental CO_2_ via carbonic anhydrase (CA) and fungal adenyl cyclase [19]–[22]. CO_2_ diffusion into or out of the cells is facilitated by its conversion to biocarbonate ions (HCO3^−^), which are utilized for several cellular processes in the cell. CO_2_-HCO_3_^−^ inter-conversion is catalyzed by CAs, which are zinc metalloenzymes and are grouped into five evolutionarily unrelated families, α, β, γ, δ, and *ε*-CA [23]–[25]. Of these, β-CA is unique to fungi and reported to be essential for fungal growth in ambient air (CO_2_ ∼ 0.036%) but not in a high CO_2_ (5%) environment [19]–[22].

In the present study, we focused on the genetic analyses of β-carbonic anhydrase (β-CA) encoded by *C. gattii CAN1* and *CAN2* as later is critical for CO_2_ sensing in a closely related pathogen *C. neoformans*. Our results provide insight into *C. gattii* adaptation for arboreal growth and the production of infectious propagules by β-CA independent mechanism (s).

## Methods

### Strains and media

The *C. gattii* strains used in this study are listed in Table 1. These strains were routinely maintained on yeast extract peptone dextrose agar (YPD) slants, and were stored in 15% glycerol at −70°C. YPD containing nourseothricin (100 µg/ml) or hygromycin B (200 µg/ml) was used to screen *can1*, and *can2* single mutant and *can1can2* double mutant strains [26]. The preparation of the various media -V8 medium for sexual (α-**a)** mating, filament agar for monokaryotic fruiting, Niger seed agar for melanin production, urea agar for urease production, and agar based Dulbecco’s modified Eagle’s (DME) medium for capsule production were used as described [27]. YPD containing menadione (3 µg/ml), or paraquat (1 mM) was used for oxidative stress, NaCl (1.4 M, and 1.8 M) for osmotic stress, and NaNO_2_ (1 mM) for nitrosative stress were prepared as described [26]. Yeast nitrogen base (YNB) broth containing various sugars was prepared as described [28]. For determination of amino acid requirements, synthetic dextrose (SD) medium containing 0.17% YNB and 1% glucose was supplemented with adenine (20 mg/l), uracil (30 mg/l), L-arginine (20 mg/l), leucine (60 mg/l), histidine (20 mg/l), tryptophane (30 mg/l). For determination of fatty acid requirements, YPD agar supplemented with palmitate (1–10 mM) or myristate (1–10 mM) and with 1% Tween 80 as surfactant was prepared as described previously [20].

**Table 1:** *Cryptococcus gattii* strains used in this study.

Strain	Genotype	Source
NIH 444 (ATCC 32609)	Wild type *MAT*α (serotype B)	American Type Culture Collection (ATCC), Manassas, VA
NIH 198	Wild type *MAT* **a** (serotype B)	Kwon-Chung K.J. (NIH, Bethesda, Maryland)
*can1-1*	*MAT*α wild type *can1::NAT*	This study
*can1-2*	*MAT*α wild type *can1::NAT*	This study
*can2*	*MAT*α wild type *can2::NAT*	This study
*can2 + CAN2*	*MAT*α wild type *CAN2*	This study
*can1can2-1*	*MAT*α wild type *can1::NAT*;*can2::HYG*	This study
*can1can2-2*	*MAT*α wild type *can1::NAT*;*can2::HYG*	This study

### Plasmids and oligonucleotides

Plasmids and oligonucleotides used in this study are listed in Table 2. The full-length *CAN1* and *CAN2* gene sequences from *C. neoformans* were BLAST searched in the NCBI database for *C. gattii* (R265) (http://www.ncbi.nlm.nih.gov/blast/Blast.cgi), which yielded R265 cont1.355, and R265 cont1.479, for *CAN1* and *CAN2*, respectively. Primers were designed to amplify approximately 1500-bp fragments of the *CAN1* and *CAN2* genes from genomic DNA of NIH 444 strain of *C. gattii*. The nucleotide sequences for the *CAN1* and *CAN2* genes from NIH 444 have been submitted to the GenBank database (*CAN1*  =  EU723699; *CAN2*  =  EU723700). The *C. neoformans* cDNA sequences from *CAN1* and *CAN2* were aligned with the *C. gattii CAN1* and *CAN2* genomic sequences, using the GAP function of the GCG Wisconsin package to obtain exon/intron boundaries. cDNA sequences for *C. gattii* retrieved through this analysis were used in multiple alignments for comparison with *C. neoformans.*

**Table 2:** Plasmids and oligonucleotides used in this study.

Plasmids	Description	Source
pCH333	*ACT1::NAT::TRP1* cloned into pCR2.1	Heitman J. (Duke University)
pJAF15	*ACT1::HYG::TRP1* cloned into pCR2.1	Heitman J. (Duke University)

Oligonucleotides	Sequences	Purpose
V1442	5’-ATCTGCAGAATTCGCCCTTA-3’	1.7-kb *NAT* amplification
V1460	5’-GAATTCGCCCTTGAAGAGAT-3’	1.7-kb *NAT* amplification
V1467	5’-CAGTCGTGGTGCTTGATTGT-3’	*CAN2* ORF
V1470	5’-ACCCAGTCCTTGATCACGTC-3’	*CAN2* ORF
V1496	5’-ACGCGGTGCATATAACCAA-3’	Diagnostic primer for *can2* single and *can1can2* double mutants
V1497	5’-CCTTCAGGCACCACTCTCAT-3’	Diagnostic primers for *can2* single and *can1can2* double mutants
V1511	5’-CCACGGAGCTCATCTTTCAT-3’	*CAN2* upstream region
V1650	5’-TCCCGCAGCCTAAGGGCGAAT TCTGCAGATCAACGTTTTCTCAGCCCTCT-3’	*CAN2* upstream region
V1514	5’-GCCACACGTCACAACTCAAA-3’	*CAN2* downstream region
V1651	5’-CTCGTTTCTACATCTCTTCAAG GGCGAATTCACCCATCATCAGGTTGAAGC-3’	*CAN2* downstream region
V1515	5’-GGTGATTGTTTCGAGTGATGA-3’	*CAN1* upstream region
V1516	5’-TCCCGCAGCCTAAGGGCGAATT CTGCAGATGAAGCGGTCCGTAGAAGGTT-3’	*CAN1* upstream region
V1517	5’-CTCGTTTCTACATCTCTTCAAGG GCGAATTCCAGCTCTCAACGTTTCTCGTC-3’	*CAN1* downstream region
V1518	5’-CAACCATGAACAGCCTTACG-3’	*CAN1* downstream region
V1609	5’-AAACTTCAAGCTTCCGCTGC-3’	Diagnostic primers for *can1* mutant screen
V1610	5’-TCTGAGGGTCTTTCCATAGC-3’	Diagnostic primers for *can1* mutant screen
V1685	5’-GGTTTATCTGTATTAACACGG-3’	1.4-kb *HYG* amplification
V1686	5’-GCTGCGAGGATGTGAGCTG-3’	1.4-kb *HYG* amplification

### Disruption of *C. gattii CAN1* and *CAN2* genes

Gene disruption was carried out as described previously [26], [29]. Disruption cassettes for *CAN1* and *CAN2* were constructed by PCR fusion [30]. In brief, upstream and downstream regions flanking the *CAN1* and *CAN2* genes (approximately 1 kb on either side) and the full-length *NAT* marker gene from pCH333 plasmid were PCR-amplified. PCR amplicons were gel-purified, added in a molar ratio of 1∶3∶1, as 5’-flanking (*CAN1* or *CAN2*):marker (*NAT*):3’-flanking (*CAN1* or *CAN2*) amplicons, followed by reaction at 94°C for 2 min, and 15 cycles at 94°C for 30 s and 58°C for 10 min to allow fusion to occur. The fusion product was used as template in conventional PCR, to obtain *can1::NAT* and *can2::NAT* alleles. The constructs were directly used to transform *C. gattii* NIH 444 wild type (WT) strain by biolistic delivery, and transformants were selected on YPD containing nourseothricin. The potential *can1* and *can2* mutants were screened by diagnostic PCR using primer pair V1609/V1610 designed from the *CAN1* flanking *NAT* gene and V1496/1497 from the *CAN2* flanking *NAT* gene. The *can1* and *can2* mutants were further confirmed for gene deletion and single integration events by reverse transcriptase (RT)-PCR and Southern blot analyses, respectively. The *can1can2* double knockout mutants were created by disruption of the *CAN2* gene in the *can1* mutant using the *can2::HYG* allele, followed by diagnostic PCR and Southern blot analyses, as described for the *can2* single mutant. Two of these clones termed *can1can2-1*, and *can1can2-2* were used for further studies.

For construction of the *CAN2* reconstituted strain, a 2.9-kb fragment containing full-length *CAN2* ORF was PCR-amplified from genomic DNA of *C. gattii* WT strain using primers V1467/V1470. The PCR fragment was cloned into pCR2.1-TOPO (Invitrogen) to yield pCR2.1-*CAN2*, and then sequenced for confirmation. The plasmid was digested with *Eco*RI, and the *CAN2* full-length fragment was biolistically transformed into the *can2* mutant, and transformants were selected on YPD medium in ambient air. Since the *can2* mutant did not grow in ambient air, clones recovered under these conditions were potential *CAN2* reconstituted strains. These transformants were patched on YPD-nourseothricin plates. Inability to grow on this medium was considered as an indication of *can2+CAN2* reconstituted strains with *CAN2* integration in the native locus, resulting in the removal of the *can2::NAT* allele. These reconstituted strains were further confirmed for single *CAN2* homologous integration event by Southern blot. One of these clones termed as *can2+CAN2* was used for further investigations.

### Analysis of nutritional requirements of *can2* mutant

Cultures grown overnight in YPD broth at 30°C with 5% CO_2_ were washed with sterile water, inoculated at OD_600_  =  0.1 in YNB containing various sugars, and incubated in ambient air with shaking for 1 week. To determine amino acid and fatty acid requirements, 5µl of serial dilutions of yeast suspension from original stock of 10^7^/ml were spotted on an appropriate medium supplemented with various amino acids or fatty acids. Cultures were incubated for 2-5 days at 30°C in ambient air (0.036% CO_2_) or in 5% CO_2_.

### Mating assays

V8 medium, buffered either with 100 mM MOPS for pH 7.0 or with sodium citrate for pH 5.0 and filament agar (pH 5.0) was used for mating and monokaryotic fruiting assays [20]. Cultures grown overnight in YPD broth at 30°C with high CO_2_ (5%) were washed twice with sterile distilled water, and were re-suspended in water at a concentration of 5 × 10^7^ cells/ml. An equal number of cells of the opposite mating type cells was mixed, and 5µl of the mixture inoculated on buffered V8 medium, and incubated at 30°C with or without CO_2_ for up to 8 weeks. For monokaryotic fruiting, 5-10µl of individually washed cells (5 × 10^7^/ml) were inoculated on filament agar and buffered V8 agar media, and incubated with or without CO_2_ for 8 weeks. Images of hyphal growth were captured with an Olympus AX70 microscope equipped with a digital camera as described previously [27].

### Assays for virulence factor expression and stress sensitivity

The *C. gattii* WT, *can1, can2* single mutants, *can1can2* double mutant, and *can2+CAN2* reconstituted strains were incubated for 16–18 hours in YPD broth at 30°C with 5% CO_2_. Cells were washed with sterile distilled water, counted, and adjusted to 10^8^/ml. Five microliters of yeast suspension were spotted on DME agar, on Christensen’s agar, and on egg yolk agar and incubated for 24–72 hours at 30°C with 5% CO_2_ for respective assessments of capsule, urease, and phospholipase production. For determination of stress sensitivity, yeast cells grown as described above were serially diluted (10^3^–10^7^), and spotted on YPD medium containing redox cycling agents menadione (3µg/ml), paraquat (1µM), sodium nitrite (0–10 mM), and sodium chloride (1–1.8 M), and incubated at 30°C with 5% CO_2_.

### Virulence assays

The pathogenic potentials of the *C. gattii* WT, *can1*, *can2* single mutants, *can1can2* double mutant, and *can2+CAN2*-reconstituted strains were assessed in a mouse model of pulmonary and systemic cryptococcosis [26], [29]. BALB/c mice (6–8 weeks) were procured from Charles River Laboratories, Inc., and procedures for safe and pain-free handling of animals were followed as per the protocol approved by the Institutional Animal Care and Use Committee (IACUC), Wadsworth Center, New York State Department of Health, Albany, NY, USA. Cultures grown overnight in YPD broth at 30°C with 5% CO_2_ were washed, and then re-suspended in sterile phosphate buffered saline (PBS), pH 7.4, at a concentration of 1 × 10^7^/ml. Group of five mice were injected intravenously with 10^6^ CFU of each strain. The animals were given food and water *ad libitum*, and were observed twice daily for any sign of distress. Mice that appeared moribund or in pain were sacrificed using CO_2_ inhalation and cervical dislocation as per the protocol approved by the Institutional Animal Care and Use Committee (IACUC), Wadsworth Center, New York State Department of Health, Albany, NY, USA. Survival data were analyzed by Kaplan-Meyer survival curve using the SAS software (SAS Institute Inc., Cary, NC, USA).

To determine the pathogenic potential of test strains in pulmonary infection, we inoculated a group of three mice with 10^5^ CFU of each strain in a volume of 30µl via nasal inhalation as previously described [26]. Animal care procedures were as per approved IACUC protocol. Animals were sacrificed after 14 days of infection, lungs and brains were removed aseptically, homogenized, serially diluted, and plated on YPD agar, and incubated at 30°C with 5% CO_2_ for CFU enumeration.

For histopathology, the left lung lobe was dissected and immersion-fixed in formalin; it was embedded and processed into paraffin blocks, sectioned at 4µm and stained with mucicarmine (Richard-Allan Scientific, Kalamazoo, MI).

## Results

### *CAN2* but not *CAN1* is a major β-CA required for *C. gattii* growth at ambient air

We identified two CA encoding genes, *CAN1* and *CAN2* in the *C. gattii* genome database for related strain R265 (www.broad.mit.edu/annotation/genome/cryptococcus_neoformans_b/Blast.html). The pair-wise comparison revealed 58% and 42% identity at nucleotide and amino acid levels. Both deduced Can1p and Can2p sequences exhibited β-CA signature motif comprising one histidine, two cysteins, and one aspartate residue critical for zinc-binding and enzyme activity. Comparison of deduced amino acid sequences of *C. gattii* Can1p and Can2p with that of *C. neoformans* Can1p and Can2p revealed them to be 89% and 97% identical, respectively, indicating that the two genes are highly conserved in *C. neoformans* and *C. gattii* [20].

**Figure 1:**
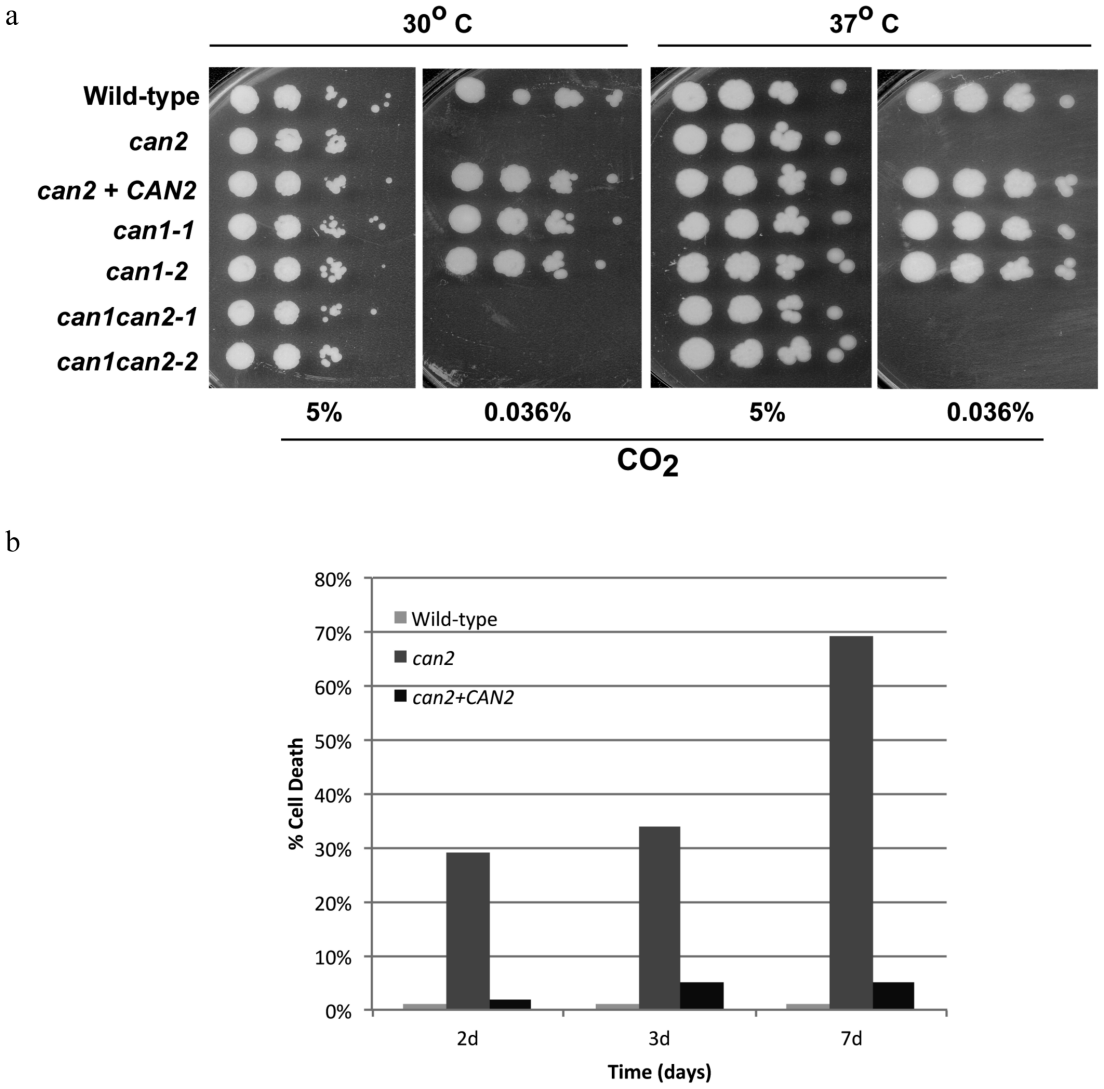
Role of β-CA in *C. gattii* growth in ambient air and in high CO_2_ environment. **(a)**. *CAN2* is major β-CA essential for *C. gattii* growth in ambient air. *C. gattii* WT and various *can* mutant strains were spotted on YPD agar and incubated at 30°C and 37°C in low CO_2_ (0.036%; ambient air) or high CO_2_ (5%) for 2–4 days. The *can2* mutant did not grow on YPD agar at either 30°C or 37°C in low CO_2_, but grew well in high CO_2_ condition. The severe growth defect phenotype of *can2* was rescued by re-introduction of WT *CAN2* allele. **(b).** *CAN2* -mediated *C. gattii* growth inhibition in ambient air is fungicidal. Approximately 100 colony forming unit (CFU) each of the *C. gattii* WT, the *can2* mutant, and the *can2+CAN2* reconstituted strain were inoculated on YPD agar, and plates were incubated for 2, 3, and 7 days at 30°C in ambient air, and then transferred to a high-CO_2_ environment. Control plates were incubated directly in high CO_2_ environment. Results were expressed as the percentage of *C. gattii* killed  =  [1- (CFU of experiment/CFU of control)] × 100. The percentage of *can2* mutant cell death was significantly higher than WT and reconstituted strains (p<0.05).

To assess the role of β-CA in *C. gattii* biology, we created *can1*, and *can2* single knockout mutants and a *can1can2* double knockout mutant through homologous integration (see method and Figure S1). The *can1* mutant did not exhibit any growth defects in either ambient air (0.036% CO_2_) or in a high-CO_2_ (5%) environment. The *can2* mutant in contrast, exhibited a severe growth defect in ambient air, but not in a high-CO_2_ environment. The *can1can2* double knockout mutants exhibited a growth phenotype similar to that of *can2* single mutant. The severe growth defect of the *can2* mutant in ambient air was rescued by re-introduction of wild-type *CAN2* allele (Fig. 1a). These results indicated that *CAN2* but not *CAN1* is essential for *C. gattii* growth in ambient air. Prolonged incubation of the *can2* mutant in ambient air was irreversibly lethal; majority of the cells could not be rescued by a shift to a high-CO_2_ environment (Fig. 1b).

### *CAN2* is critical for fatty acid biosynthesis but not required for adenyl cyclase (*CAC1*) gene expression

**Figure 2:**
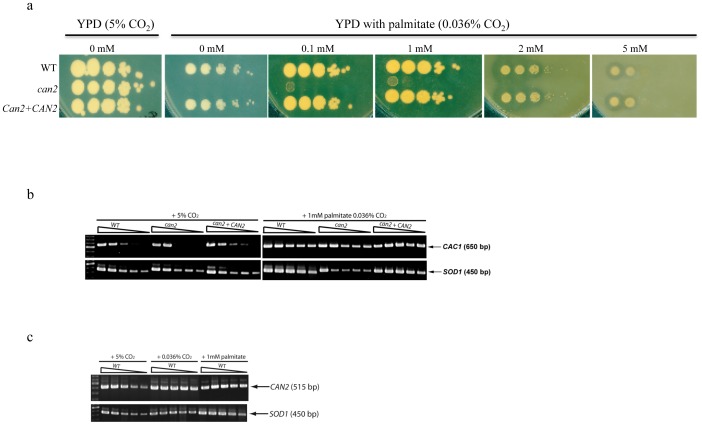
*CAN2* is critical for fatty acid biosynthesis but not essential for adenyl cycalse (*CAC1*) gene expression. **(a)**. Palmitic acid barely restored *can2* mutant growth in ambient air. *C. gattii* strains grown overnight in high CO_2_ were collected, washed, serially diluted, and spotted on YPD medium containing palmitate with 1% Tween 80 as surfactant. Cells were incubated at 30°C in ambient air for 5 days. The growth defect of *can2* mutant was barely rescued in the presence of low but not high concentration of palmitate in ambient air. The halo surrounding the growth patches of WT and reconstituted strains reflects efficient utilization of fatty acids from media. **(b)** Semi-quantitative RT-PCR confirmed *CAC1* gene expression is independent of *CAN2*. *C. gattii* WT, *can2* mutant, and *can2+CAN2* reconstituted strain were grown in YPD broth in high CO_2_ or in YPD broth containing 1 mM sodium palmitate in ambient air, for 3 days at 30°C. Total RNA was isolated and reverse transcribed (cDNA) with 100-ng aliquots in 1∶10 serial dilutions. *SOD1* was used as a loading control. **(c)** Semi-quantitative RT-PCR confirmed *CAN2* gene expression is not regulated by CO_2_. *CAN2* transcript in total RNA was determined from *C. gattii* WT strain grown in various conditions as indicated. *SOD1* was used as a loading control.

We reasoned that the inability of the *can2* mutant to grow in air could be due to limiting amounts of bicarbonate, a critical substrate required for the synthesis of several cellular carboxylases important in metabolism [31]. Bicarbonate is also a critical substrate for *CAC1* gene activation, and that in turn leads to the synthesis of cAMP, a ubiquitous second messenger that regulates a large variety of essential physiological processes [21], [22]. Interestingly, addition of exogenous cAMP (2–10 mM) or sodium bicarbonate (1–10 mM) either singly or in combination, failed to complement the growth defect of the *can2* mutant in ambient air. Similarly, addition of various cellular metabolites and carbon sources, including citrate, succinate, oxalaacetate, malate, α-ketoglutarate failed to complement the growth defect of the *can2* mutant (data not shown). In contrast to the report published for *C. neoformans*, the growth defect of the *can2* mutant was barely rescued by addition of exogenous fatty acids, 0.1 mM and 1 mM palmitate (Fig. 2a), indicating that *CAN2* is essential for fatty-acid biosynthetic processes in ambient air in *C. gattii*. We observed a clear zone surrounding the colonies of *C. gattii* WT and *can2+CAN2* reconstituted strains (2 mM and 5 mM palmitate) (Fig. 2a). This might be due to the fact that WT and reconstitute strains were able to utilize fatty acids from media resulting in clear zone surrounding the growth.

To explore the link between *CAN2* and *CAC1*, RNA was extracted from WT, *can2* mutant, and *can2+CAN2* reconstitute strain grown for 3 days in ambient air in the presence of 1mM sodium palmitate or in a high-CO_2_ environment. We found that the *can2* mutant remains viable (100%) but do not multiply in the presence of 1 mM sodium palmitate in ambient air for up to 4 days (data not shown). Semi-quantitative RT-PCR revealed that *CAC1* transcript was expressed with or without CO_2_ in both *can2* mutant and in the WT strain and also *CAC1* expression appeared to be marginally induced without CO_2_, which was consistent for WT, *can2* mutant and *can2+CAN2* reconstituted strains (Fig. 2b). These results indicated that *CAC1* expression is independent of *CAN2*, in other words, *CAN2* is not required for *CAC1* expression. Also, semi-quantitative RT-PCR analysis of *CAN2* transcript in *C. gattii* WT revealed similar expression pattern in both ambient air or in high CO_2_ environment. These results indicated that *CAN2* gene expression is not regulated by CO_2_ (Fig. 2c).

### CO_2_ is a powerful inducer of monokaryotic hyphae development in *C. gattii*

**Figure 3:**
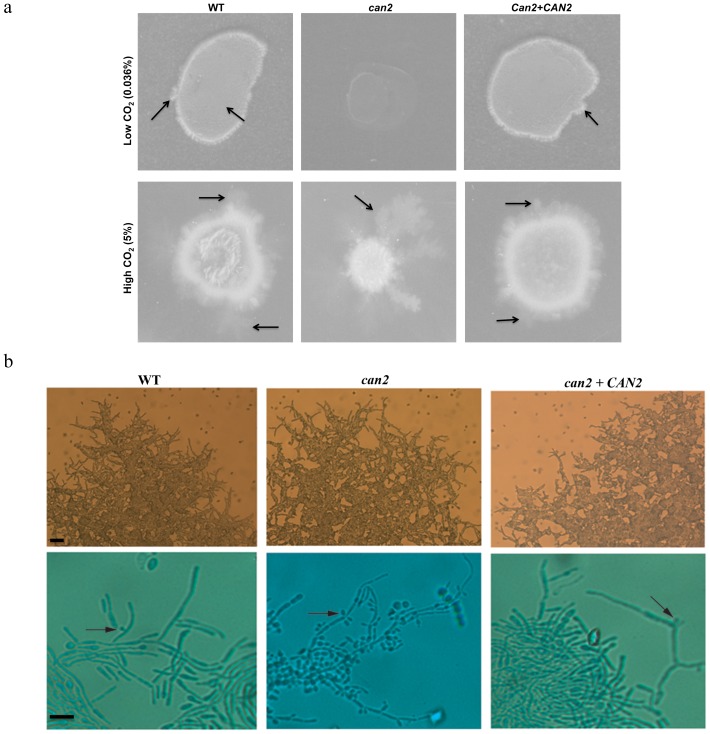
CO_2_ is a powerful inducer of monokaryotic hyphae development in *C. gattii*. *C. gattii* strains were individually cultured on filament agar and hyphae development was assessed macroscopically at 8 weeks-post incubation. **(a)** Upper panel- Few filamentous projections (arrow) seen at the edge of the colonies of WT and *can2+CAN2* reconstituted strains. No growth of *can2* mutant in ambient air (low CO_2_). Lower panel-Robust filamentation (arrow) in the presence of high CO_2_ with dense and long hyphal extension in *can2* mutant. **(b)** Upper panel - Light microscopic analyses of hyphae development (magnification, × 100) in WT, *can2,* and *can2+CAN2* strains in the presence of high CO_2_. Lower panel - Filamentous growth on the edge of the colony were carefully removed, mounted on lactophenol cotton blue and gently pressed and photographed (magnification, × 200). Filaments bearing blastospores (arrows) seen in all the strains except that *can2* mutant revealed more blastospores compared to the WT and *can2+CAN2* reconstituted strains.

Mating is an important process by which *Cryptococcus* generates filaments and spores that might be important in its ecological fitness. It is clear that *C. gattii* associates with various plant species in nature [1]. However, it is not clear how this fungus survives and propagates on plant substrates. Since most of the plants utilize CO_2_ for photosynthesis, and they possess a CO_2_ concentration mechanism through RubisCO, an enzyme specifically found in chloroplasts of bundle sheath cells [18], we asked whether high CO_2_ induces mating and hyphae development in *C. gattii*. The *C. gattii* WT, *can1* and *can2* single mutants, *can1can2* double mutant, and *can2+CAN2* reconstitute strains were inoculated on filament agar and V8 agar for monokaryotic and sexual mating. The inoculated plates were incubated in ambient air or in high CO_2_. To our surprise, we found that *C. gattii* WT strain undergoes hyphae development as part of monokaryotic fruiting more vigorously in high CO_2_ than in ambient air (Fig. 3a). Filaments on the edges of *C. gattii* WT growth appeared as early as 1-week post-incubation under high CO_2_, compared to 4-weeks post-incubation under low CO_2_. The *can2* but not *can1* mutation caused further enhancement of filamentation as judged by long and dense filaments on the colony edges (Fig. 3a; lower panel). Light microscopic mounts of these filamentous projections from the WT as well from the *can2* mutant revealed hyphae and blastospores but not basidiospores. The *can2* mutant hyphae harbored several blastospores, whereas the WT and *can2+CAN2* reconstituted strains harbored few blastospores (Fig. 3b; lower panel). The topology of hyphae harboring blastospores was consistent with our earlier report where these structures were analyzed by scanning electron microscope [27]. The monokaryotic filamentation was also observed on V8 agar at pH 7.0 with or without CO_2_ but not at pH 5.0. However, filamentation was not as robust as on filament agar (data not shown).

**Figure 4:**
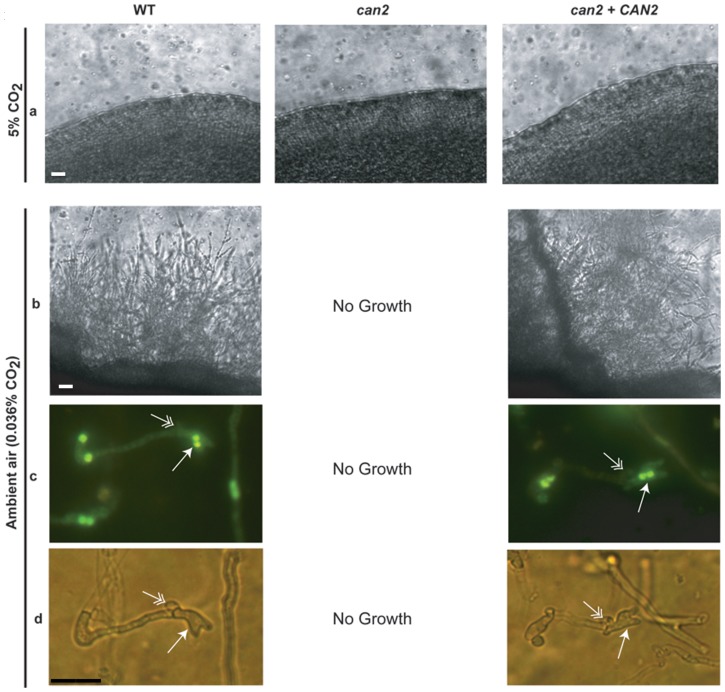
High CO_2_ inhibits sexual mating in *C. gattii* and *CAN2* does not rescue this inhibition. An equal number of *C. gattii* WT, *can2* mutant, and *can2+CAN2* reconstituted strains were mixed with the *MAT* **a** strain (NIH 198), inoculated on V8 agar and incubated in high (5%) or low (0.036%) CO_2_ and mating was assessed at 8 weeks post-incubation. **(a)** Light microscopic analyses (magnification, × 100) of the representative edges of the mating patches showing no filamentation in high CO_2_. **(b)** Light microscopic analyses (magnification, × 100) of the representative edges of the mating patches with extensive filamentation in low CO_2_ (ambient air). No growth of *can2* mutant in ambient air. **(c)** Filamentous growth on the edge of the colony was carefully removed, and stained with SYTOX Green and assessed under fluorescent microscope (magnification, × 400). Filaments showing characteristic fused clamp connection (single arrow) and pairs of nuclei (double arrows) upon mating of WT (α) × WT (**a**), and *can2+CAN2* (α) × WT (**a**). No growth of *can2* mutant was evident in ambient air. (**d**) Light microscopy of same structures as shown in c (magnification, × 400).

In contrast, high CO_2_ completely suppressed sexual mating (α-**a**) in *C. gattii* WT, *can2* mutant, and *can2+CAN2* reconstituted strains, as no filamentation on the edges of the colonies was observed even after 8 weeks of incubation on V8 agar medium adjusted to either pH 5 or 7 (Fig. 4a). *C. gattii* WT and *can2+CAN2* reconstituted strains showed robust sexual mating under low CO_2_ in V8 agar medium adjusted to pH 7.0 (Fig. 4b), but not to pH 5.0 (data not shown). Hyphae cells produced during sexual mating contained two nuclei (single arrow) that were linked by fused clamp (double arrow) connections (Fig. 4c & 4d). It should be pointed out here that we used only unilateral crossing in which WT *MAT* **a** strain (NIH 198) was used as the opposite mating partner in the sexual mating assay. Overall, these results indicated that high CO_2_ is a powerful inducer of monokaryotic hyphae differentiation but not in sexual mating providing important distinction in these two developmental programs in *C. gattii*.

### β-CA activity is not required for the expression of *C. gattii* virulence repertoire

**Figure 5:**
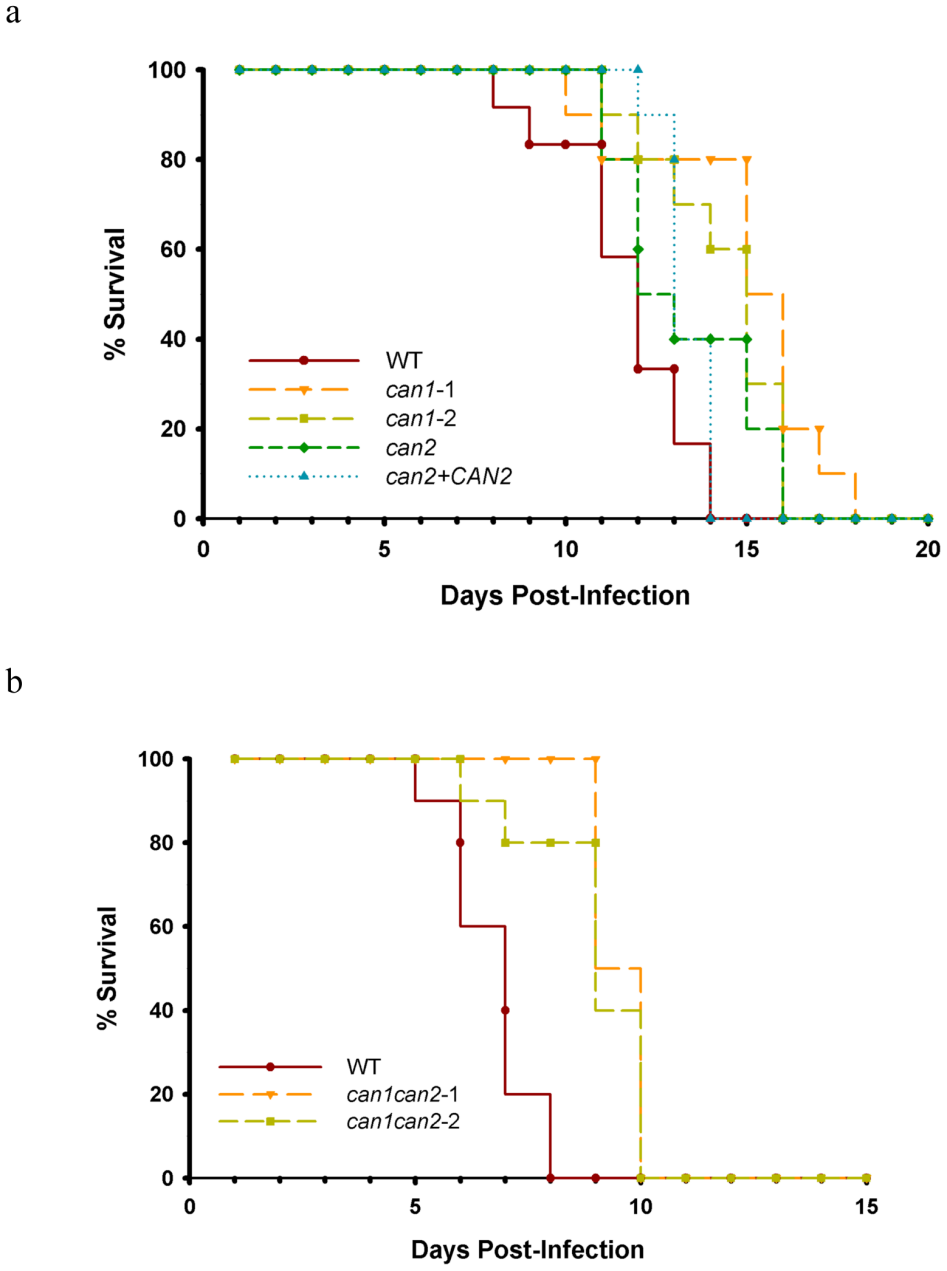
β-CA activity is dispensable for *C. gattii* pathogenesis. **(a-b)** Systemic cryptococcosis model: The WT, *can1, can2* single mutants, *can1can2* double mutants and *can2+CAN2* reconstituted strains grown overnight in YPD broth in high CO_2_ were washed with PBS, and counted, and a 100µl suspension containing 10^6^ cells was injected intravenously into 5–6 weeks old BALB/c mice (5 mice/group). Mice were monitored twice daily and sacrificed if any symptoms of distress were apparent. No significant difference on survival rate of mice infected with WT or mutant strains observed (p>0.05).

Since β-CA activity was found to be essential for *C. gattii* growth in ambient air, we asked whether this enzyme is required for *C. gattii* virulence factor expression, and furthermore, for disease development in mammalian hosts. The *can1* and *can2* single mutants, as well *can1can2* double mutant strains expressed major virulence factors (melanin, capsule, phospholipase, urease) at levels comparable to those for the WT strain, in a high-CO_2_ environment. Similarly, mutants did not exhibit any altered sensitivity to oxidative, osmotic or nitrosative stress (Figure S2). These results indicated that β-CA activity is neither required for general stress response nor for the expression of virulence traits, at least when CO_2_ is in abundance. Furthermore, β-CA activity is not essential for *C. gattii* to induce disease in mammalian host. Mice infected intravenously with the *can1* or *can2* single mutant, or *can1can2* double mutant strains manifested severe disease similarly to mice infected with the WT strain (Fig. 5a & 5b).

**Figure 6:**
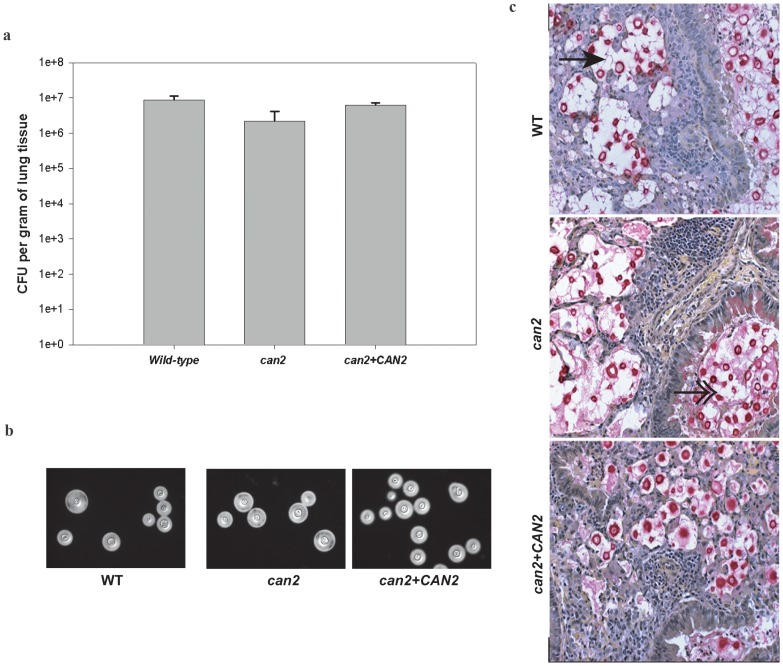
β-CA activity is dispensable for *in vivo* colonization. **(a)** Organ load determination: BALB/c mice (6–8 weeks) were infected intra-nasally with 10^5^ yeast cells of the WT, *can2,* or *can2+CAN2* strain (3 mice/strain). After 14 days, mice were sacrificed, and their lungs were removed aseptically, weighed, homogenized, and diluted in PBS, and cultured on YPD agar in high CO_2_ for CFU enumeration. Results were expressed as CFU per gram of tissue. No significant difference in organ load of WT or *can2* mutant was observed (p>0.05). **(b)** *In vivo* capsule production: WT, *can2,* and *can2+CAN2* strains recovered from mice lungs were visualized with India ink (magnification, × 100). **(c)** Histopathological examination of lungs: Left lung lobes from mice infected with the WT, the *can2* mutant, or the *can2+CAN2* reconstituted strain for 14-days were fixed, sectioned, and stained with Mayer’s mucicarmine. Alveoli (single arrow) and airways (double arrow) showed the presence of numerous organisms for each of the infecting strain and similar tissue response was noted for WT, *can2* and *can2*+*CAN2*.

Given the importance of gaseous exchange in the lungs, with the high oxygen content in the terminal alveoli, as well the lungs’ vigorous defense mechanisms against pathogens, we probed if *can2* mutant is able to colonize the lungs as efficiently as the WT strain. The organ load experiment revealed that the fungal burden imposed by the *can2* mutant was almost as high as the burden imposed by the WT strain (Fig. 6a). Also, the *can2* mutant was able to produce capsule in the lungs as large as those produced by the WT strain (Fig. 6b). Furthermore, histopathological examinations of lungs infected with *can2* mutant or the WT strain revealed similar tissue responses, including severe and diffuse interstitial pneumonia, and the presence of numerous organisms in the alveoli and airways (Fig. 6c). Altogether, these results confirmed that *CAN2* deletion has no influence on *C. gattii* virulence traits and pathogenesis, in agreement with previous findings for *C. neoformance* and *Candida albicans* [20], [21].

## Discussion

The present study revealed that high CO_2_ strongly induced monokaryotic hyphae development in *C. gattii* while it completely repressed sexual (α-**a**) hyphae development, indicating an important distinction in environmental responses by theses two developmental programs. Considering the fact that *C. gattii* grows on plants known to concentrate CO_2_ through RuBisCO [32], the observed association between high CO_2_ and morphological transition in *C. gattii* indicates an ecological adaptation for survival and propagation in nature.

Nitrogen starvation, water deprivation and high temperature have been linked to monokaryotic fruiting in *C. neoformans* [16], [17]. Also, darkness is an additional factor associated with hyphae production and fruiting structures in *C. neoformans* [33]. We have now added high CO_2_ (5%) to this list as it strongly induced hyphae development in *C. gattii*; filamentation was discernable as early as 1-week post incubation in high CO_2_ compared to its appearance at 4 weeks in a low-CO_2_ (ambient air) environment. Recently, CO_2_ has also been shown to be powerful inducer of filamentation in *C. albicans* that requires *CAC1* but bypasses Ras [21]. *CAC1* activation requires both bicarbonate and G proteins in *C. albicans* as well as in *C. neoformans* [21], [22], [34]. Interestingly, we did not find any link between *CAN2* and *CAC1* as *can2* mutant produced equivalent amount of *CAC1* transcript as the WT strain. Additionally, *CAC1* transcript was induced more in ambient air than in high CO_2_ while opposite was true for hyphae development where high CO_2_ served as powerful inducer. These results indicate that *C. gattii CAC1* may not be directly involved in CO_2_-induced monokaryotic hyphae development as opposed to its critical role assessed in sexual mating [34]. These results support the hypothesis that there are probably different signaling pathways in the development of hyphal projection, a prerequisite for spore formation in monokaryotic fruiting and sexual mating. The search of *C. gattii* database for a related strain R265 (http://www.broad.mit.edu) revealed single copy of *CAC1* gene as reported earlier for *C. neoformans* [34]. Interestingly, we found that *can2* mutant undergoes robust monokaryotic filamentation with blastospore formation indicating that either *CAN2* serves as a repressor, or certain threshold levels of CO_2_-HCO_3_^−^ interconversion is critical in this developmental pathway.

We also found that *CAN2,* but not *CAN1,* was essential for *C. gattii* growth under ambient air (0.035% CO_2_). In this regard, *C. gattii* is similar to its closely related species *C. neoformans* where *CAN2* was major β-CA for growth under ambient air [20]–[22]. The precise mechanism for observed growth defects of *C. gattii can2* mutant in ambient air is not clear at present but defective fatty acid biosynthesis might be partially responsible, consistent with earlier report for *C. neoformans* [20]. Since *CAN2* was dispensable for survival, proliferation, and lethality during intravenous and intranasal infection, its role in *C. gattii* pathogenesis appeared to be redundant.

Although very little is known about morphological forms of *C. gattii* in nature, a hyphal phase appears to be an integral part of *C. gattii* biology. The recent outbreak of *C. gattii* on Vancouver Island revealed that the fungus inhabits several tree species (Douglas fir, alder, maple, and Garry oak) [1], [4], [9]. The Vancouver Island air samples contain particles of 1–2µm in diameter, a size consistent with spores [1]. Also, all of the isolates from this outbreak belonged to *MAT*α mating type, further bearing out the predominant mode of reproduction possibly through monokaryotic fruiting. Additionally, the endemic nature of *C. gattii* in Australia, majority of Australian isolates being sterile, and their well-known association with *Eucalyptus* trees strongly suggest that the monokaryotic fruiting might be the driving force for the survival and propagation of *C. gattii* in nature [35], [36]. Although, mixed populations of *MAT*α and *MAT* **a** strains of *C. gattii* have been identified colonizing hollows in *Eucalyptus* trees in Australia [37]–[40], no meiotic recombination has been detected in isolates recovered from these hollows; thus monokaryotic fruiting could still be the main mode of propagation of *C. gattii* in nature.

In summary, we have demonstrated that high CO_2_ conditions induced robust development of monokaryotic hyphae and spores in *C. gattii*. Conversely, high CO_2_ completely repressed hyphae development in sexual mating. Both *CAN1* and *CAN2* were dispensable for CO_2_ induced morphogenetic transitions and expression of pathogenic traits. Further investigations are warranted to dissect CO_2_-mediated signaling pathways to determine relevant sensor(s) required for monokaryotic fruiting.

## Supporting Information

Figure S1 **Characterization of** ***can1*** **, and** ***can2*** **single knockout mutants,** ***can1can2*** **double knockout mutant, and** ***can2+CAN2*** **reconstituted strains.** **(a-b)** Diagnostic PCR and Southern hybridization analysis for *can1* mutants: (a) Primers (V1609/v1610) designed from the *CAN1* flanking *NAT* gene amplified 1.7-kb PCR product from the genomic DNA of *C. gattii* WT and 3.0-kb amplicon from the genomic DNA of *can1*-1 and *can1*–2 mutants obtained through two independent transformation events. (b) Genomic DNA was digested with *Sac* I (cuts once within *CAN1* gene) and probed with 612-bp PCR product amplified from *CAN1* ORF. The *C. gattii* WT produced 1.4-kb band, while both *can1*-1 and *can1*-2 mutants produced 3.3-kb bands. **(c-e)** Diagnostic PCR, RT-PCR, and Southern hybridization analyses of *can2* mutant and *can2+CAN2* reconstituted strains. (c) Primers (V1496/V1497) designed from the *CAN2* flanking *NAT* gene amplified 1.4-kb PCR product from the genomic DNA of *C. gattii* WT and *can2+CAN2* reconstituted strains while same primer set produced 2.9-kb PCR product from the genomic DNA of *can2* mutant. (d) Total RNA was isolated, reverse transcribed to cDNA and amplified with primers (V1600/V1532) directed against *CAN2* or primers (V548/V549) directed against *SOD1*. RT-PCR products were fractionated by electrophoresis in a 1% agarose gel and stained with ethidium bromide. *C. gattii* WT and *can2+CAN2* reconstituted strains yielded 515-bp *CAN2* transcript while *can2* mutant did not. *SOD1* transcript served as a loading control. (e) Genomic DNA from *C. gattii* WT, *can2* mutant, and *can2+CAN2* reconstituted strains were cut with *Hind* III (non-cutter within *CAN2* gene), and probed with 372-bp PCR product amplified from the *CAN2* gene. The *C. gattii* WT and *can2+CAN2* reconstituted strains produced 3.0-kb band while *can2* mutant produced 4.5-kb band. **(f-g)** Diagnostic PCR and Southern hybridization analyses of *can1can2* double knockout strains: For creation of *can1can2* double knockout strain, *CAN2* gene was disrupted in *can1* mutant using *can2:HYG* allele. (f) Primers (V1496/V1497) yielded 1.4-kb amplicon from the genomic DNA of *C. gattii* WT as shown in figure C while same primer pair yielded 3.2-kb amplicon from the genomic DNA of *can1can2* double knockout strains. (g) Genomic DNA from *C. gatti* WT, *can1can2*-1, and *can1-can2*-2 double knockout mutants were cut with *Hind* III and probed with *CAN2* PCR product. The *C. gattii* WT produced 3.0-kb band, while both *can1can2* double knockout mutants produced 4.9-kb bands.
(TIF)

Figure S2 **β-CA activity is dispensable for virulence factor production and for various stresses in** ***C. gattii*** **.** WT and various *can* mutant strains were grown overnight at 30°C in 5% CO_2_, washed, and adjusted to OD_600_  = 1.0. The 10-fold serial dilutions were prepared and 4µl of each dilution was spotted on YPD alone, YPD containing NaNO_2_ (nitrossative), NaCl (osmotic), menadione and paraquat (oxidative) and incubated at 30°C for 72 h. Also assessed were the production of melanin (Niger seed agar), urease (Christensen agar), phospholipase (egg-yolk agar) and capsule (DME agar). Mutant strains neither exhibited any altered sensitivity to stress nor were defective in the production of major virulence factors.
(TIF)
