# Supplementary figures and images for: Carbon Dioxide is a Powerful Inducer of Monokaryotic Hyphae and Spore Development in Cryptococcus gattii and Carbonic Anhydrase Activity is Dispensable in This Dimorphic Transition
Source: PLoS One. 2014 Dec 5;9(12):e113147. doi: 10.1371/journal.pone.0113147 (PMC4257545; doi:10.1371/journal.pone.0113147)

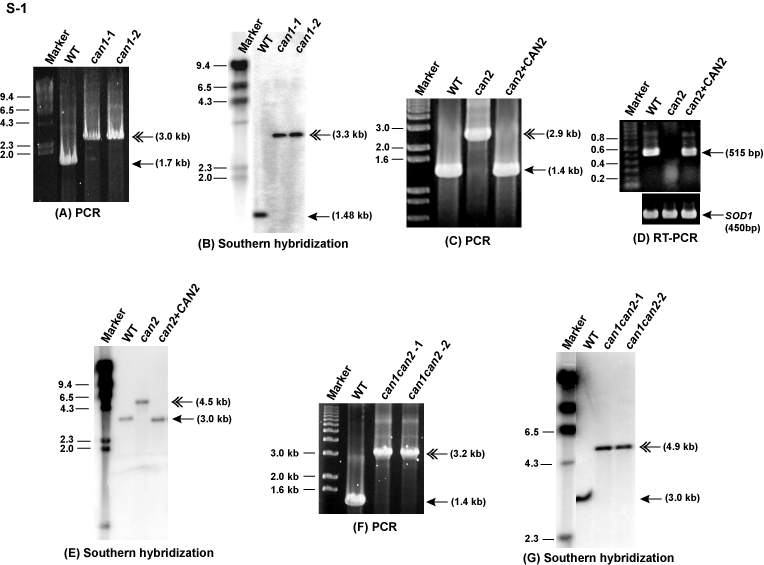

Supplement: Figure S1 — Characterization of can1 , and can2 single knockout mutants, can1can2 double knockout mutant, and can2+CAN2 reconstituted strains. (a-b) Diagnostic PCR and Southern hybridization analysis for can1 mutants: (a) Primers (V1609/v1610) designed from the CAN1 flanking NAT gene amplified 1.7-kb PCR product from the genomic DNA of C. gattii WT and 3.0-kb amplicon from the genomic DNA of can1-1 and can1–2 mutants obtained through two independent transformation events. (b) Genomic DNA was digested with Sac I (cuts once within CAN1 gene) and probed with 612-bp PCR product amplified from CAN1 ORF. The C. gattii WT produced 1.4-kb band, while both can1-1 and can1-2 mutants produced 3.3-kb bands. (c-e) Diagnostic PCR, RT-PCR, and Southern hybridization analyses of can2 mutant and can2+CAN2 reconstituted strains. (c) Primers (V1496/V1497) designed from the CAN2 flanking NAT gene amplified 1.4-kb PCR product from the genomic DNA of C. gattii WT and can2+CAN2 reconstituted strains while same primer set produced 2.9-kb PCR product from the genomic DNA of can2 mutant. (d) Total RNA was isolated, reverse transcribed to cDNA and amplified with primers (V1600/V1532) directed against CAN2 or primers (V548/V549) directed against SOD1. RT-PCR products were fractionated by electrophoresis in a 1% agarose gel and stained with ethidium bromide. C. gattii WT and can2+CAN2 reconstituted strains yielded 515-bp CAN2 transcript while can2 mutant did not. SOD1 transcript served as a loading control. (e) Genomic DNA from C. gattii WT, can2 mutant, and can2+CAN2 reconstituted strains were cut with Hind III (non-cutter within CAN2 gene), and probed with 372-bp PCR product amplified from the CAN2 gene. The C. gattii WT and can2+CAN2 reconstituted strains produced 3.0-kb band while can2 mutant produced 4.5-kb band. (f-g) Diagnostic PCR and Southern hybridization analyses of can1can2 double knockout strains: For creation of can1can2 double knockout strain, CAN2 gene was disrupted in can1 mutant using can [file pone.0113147.s001.tif]

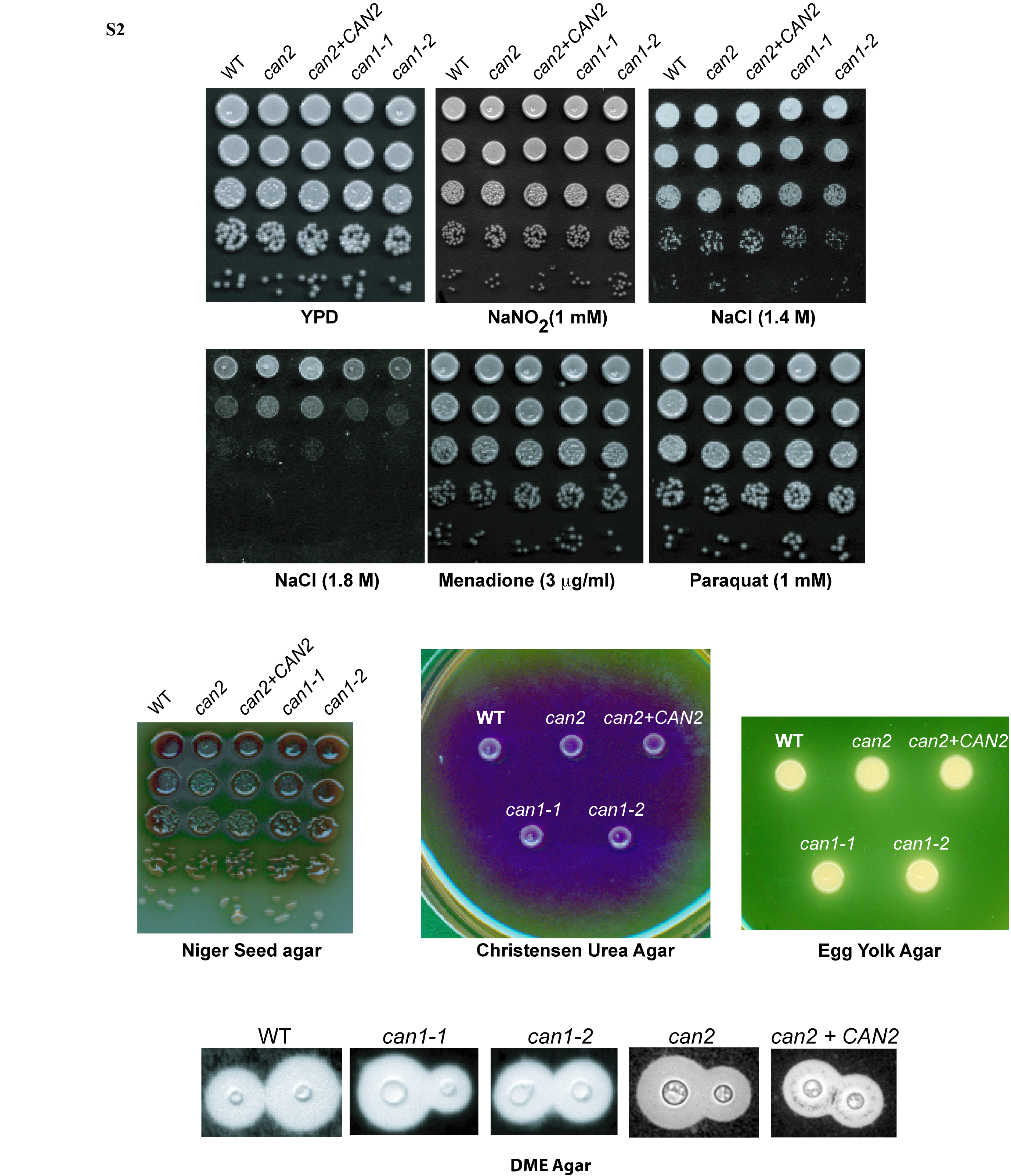

Supplement: Figure S2 — β-CA activity is dispensable for virulence factor production and for various stresses in C. gattii . WT and various can mutant strains were grown overnight at 30°C in 5% CO2, washed, and adjusted to OD600 = 1.0. The 10-fold serial dilutions were prepared and 4µl of each dilution was spotted on YPD alone, YPD containing NaNO2 (nitrossative), NaCl (osmotic), menadione and paraquat (oxidative) and incubated at 30°C for 72 h. Also assessed were the production of melanin (Niger seed agar), urease (Christensen agar), phospholipase (egg-yolk agar) and capsule (DME agar). Mutant strains neither exhibited any altered sensitivity to stress nor were defective in the production of major virulence factors. (TIF) [file pone.0113147.s002.tif]
